# Supplementary figures and images for: Quantification of metabolic niche occupancy dynamics in a Baltic Sea bacterial community
Source: mSystems. 2023 May 31;8(3):e00028-23. doi: 10.1128/msystems.00028-23 (PMC10312292; doi:10.1128/msystems.00028-23)

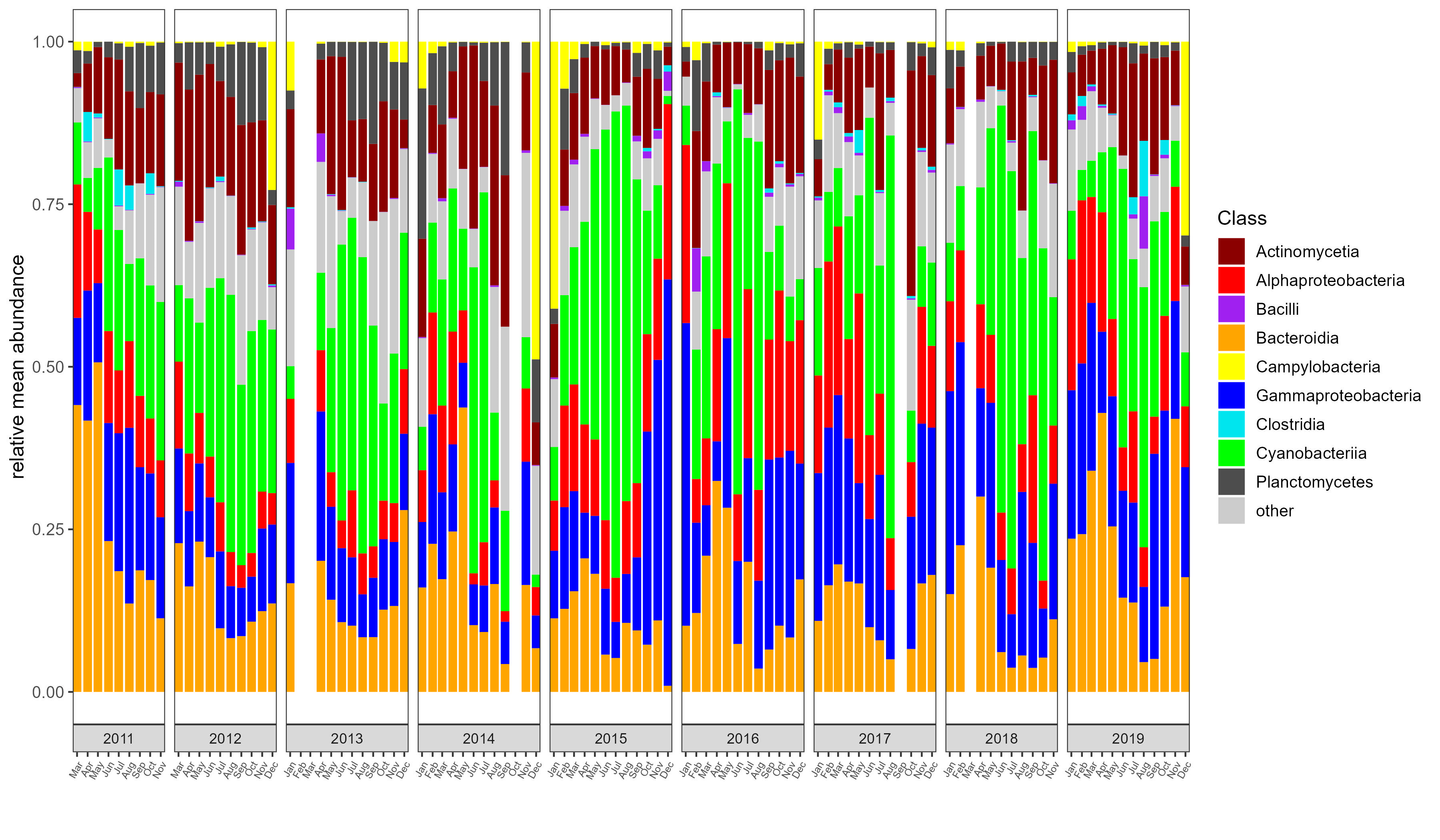

Supplement: FIG S1 — Relative mean abundances of classes that map to the genomes obtained from amplicon sequencing data over the whole sampling period. Taxonomic classes are color-coded. [file msystems.00028-23-s0001.tif]

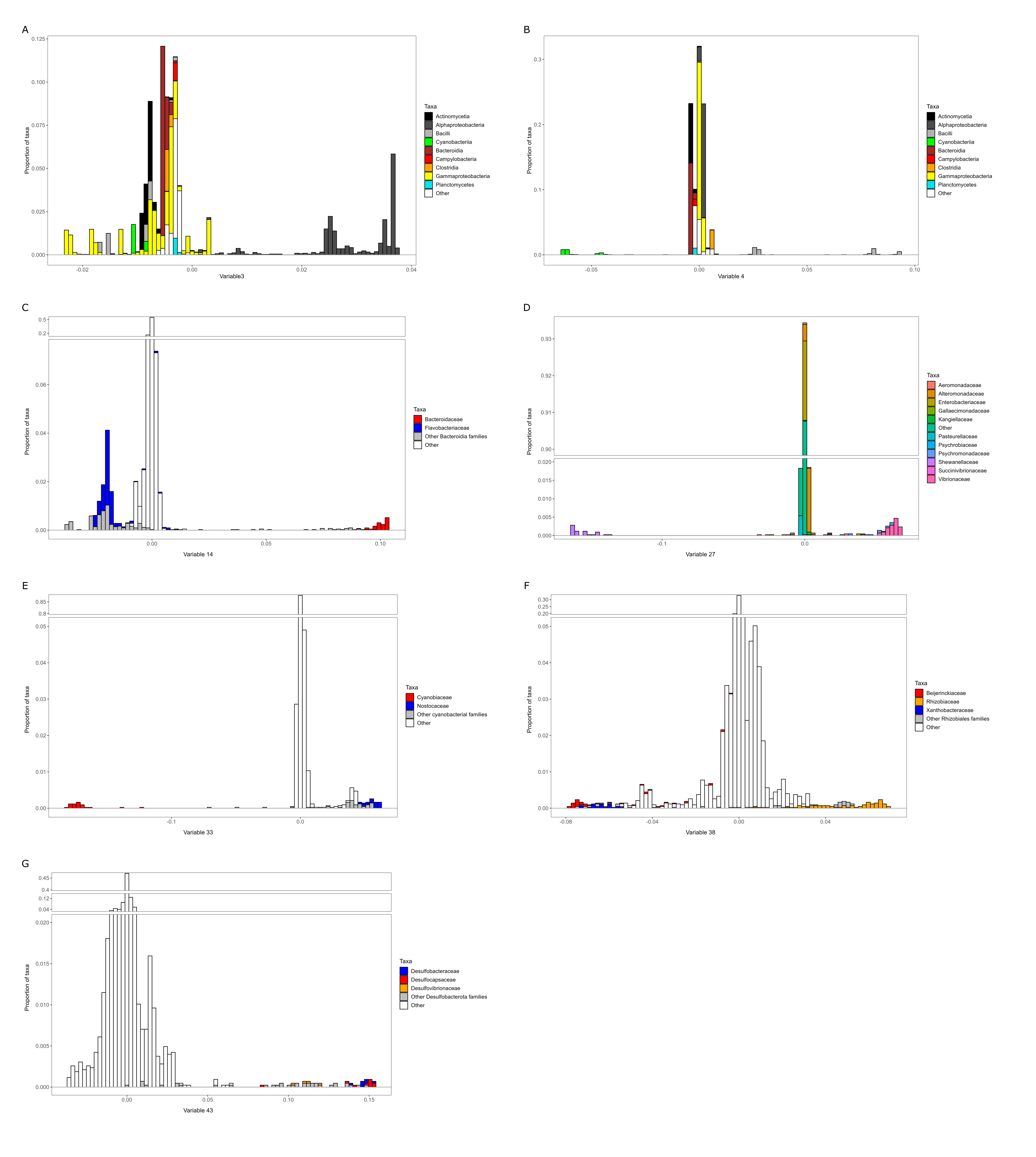

Supplement: FIG S2 — The ordering of taxa defined by variable 3 entries (A), variable 4 entries (B), variable 14 entries (C), variable 27 entries (D), variable 33 entries (E), variable 38 entries (F), variable 43 entries (G), from negative to positive (left to right). The taxonomic compositions corresponding to variable entries are shown for each of 80 equally spaced bins. [file msystems.00028-23-s0002.tif]

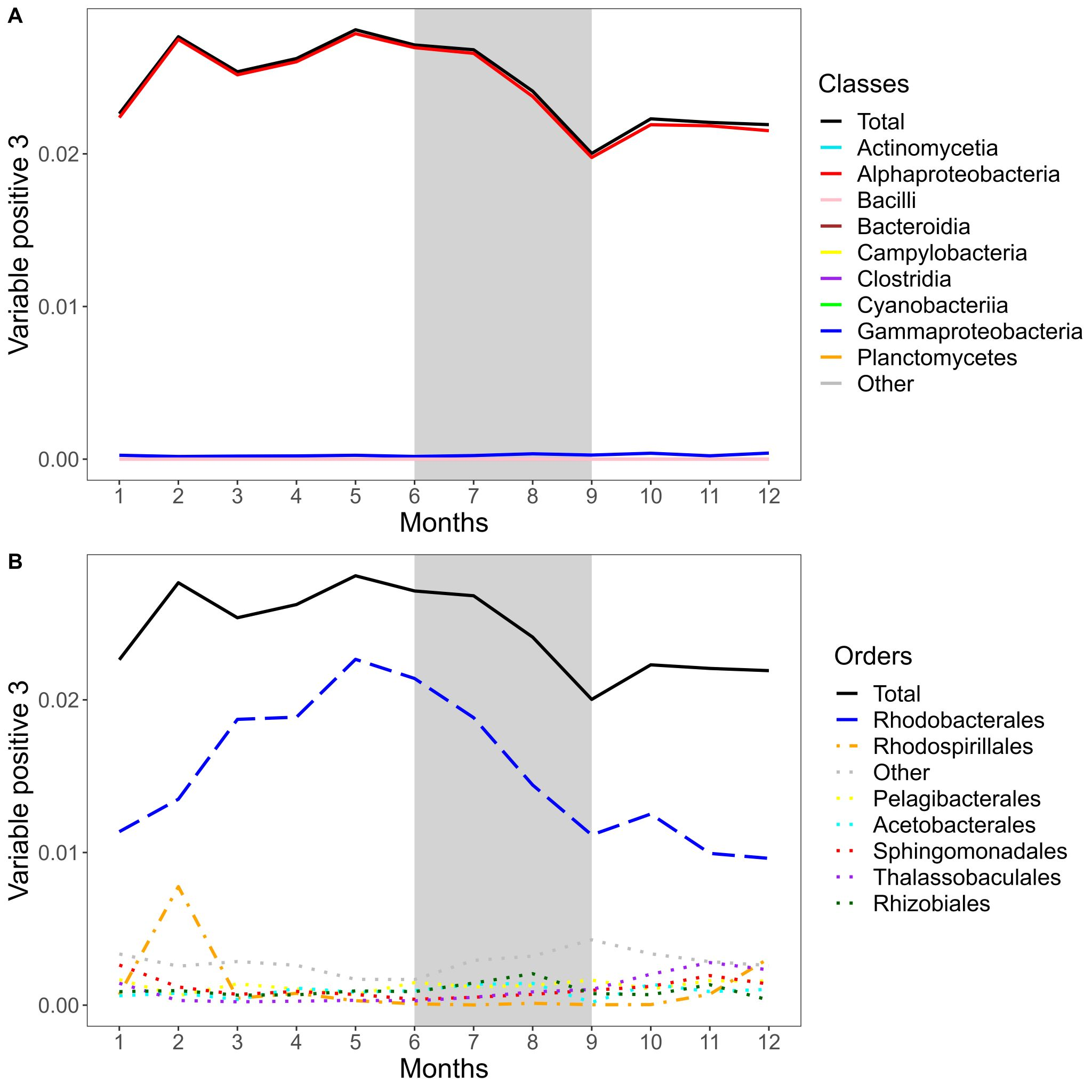

Supplement: FIG S3 — Abundance-weighted mean values of inferred ability of utilizing a variety of carbon sources over the yearly cycle. Summer months are indicated by a gray background. Taxonomic class (A) and taxonomic orders (B) are color-coded. [file msystems.00028-23-s0003.tif]

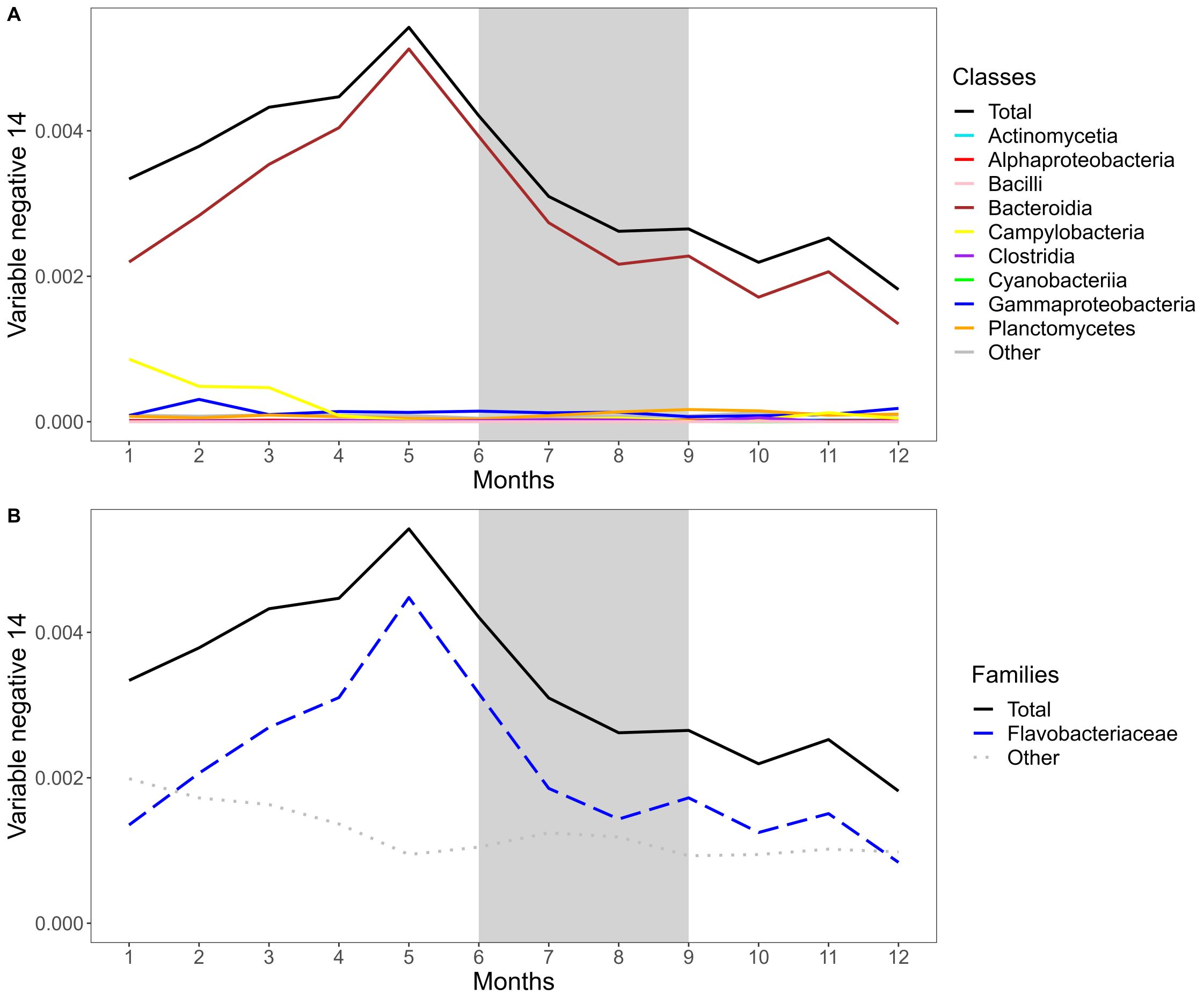

Supplement: FIG S4 — Abundance-weighted mean values of inferred ability of degrading complex polysaccharides over the yearly cycle. Summer months are indicated by a gray background. Taxonomic class (A) and taxonomic families (B) are color-coded. [file msystems.00028-23-s0004.tif]

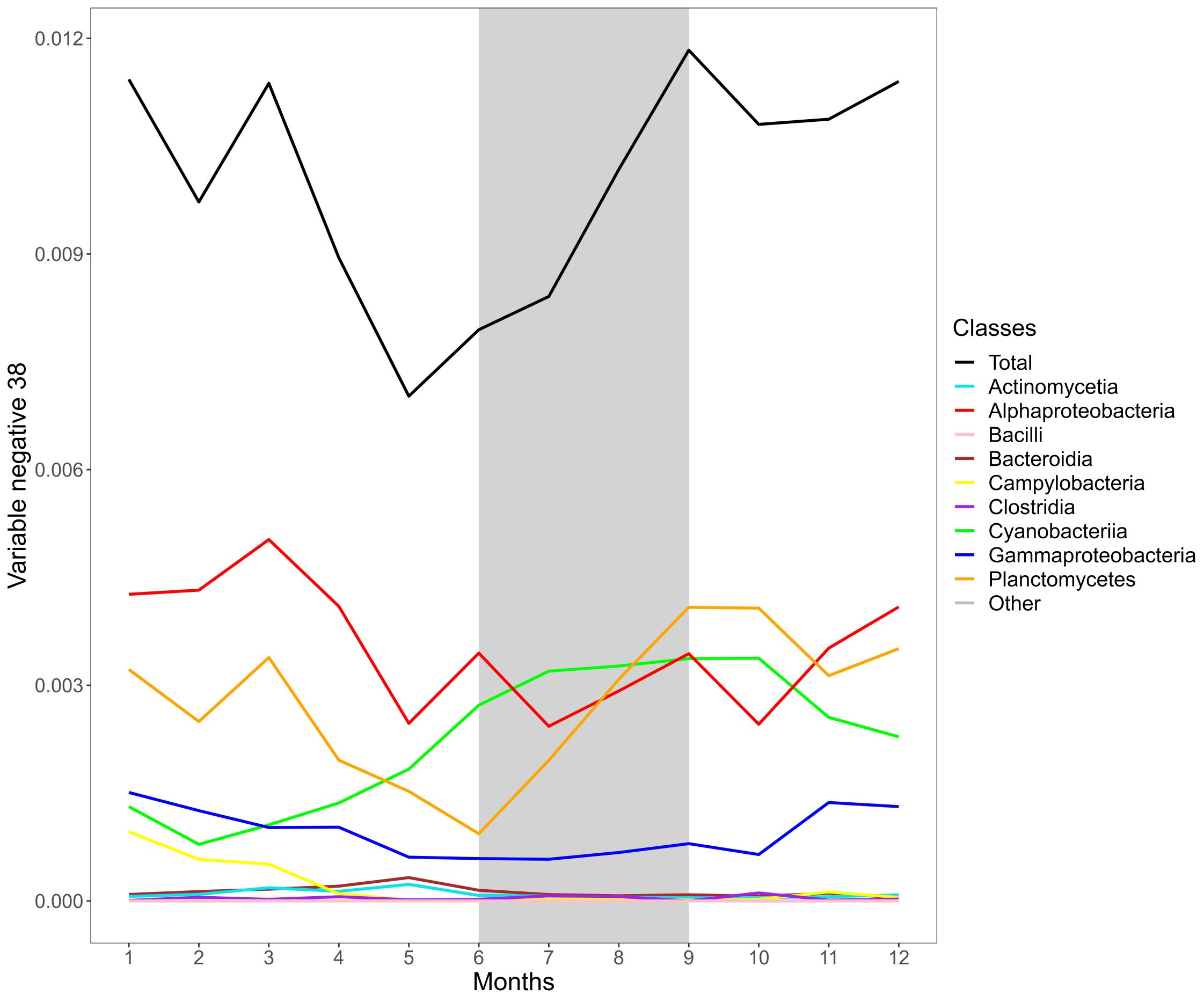

Supplement: FIG S5 — Abundance-weighted mean values of inferred ability of oxidizing methyl groups and C1 compounds over the yearly cycle. Summer months are indicated by a gray background. Taxonomic class is color-coded. [file msystems.00028-23-s0005.tif]

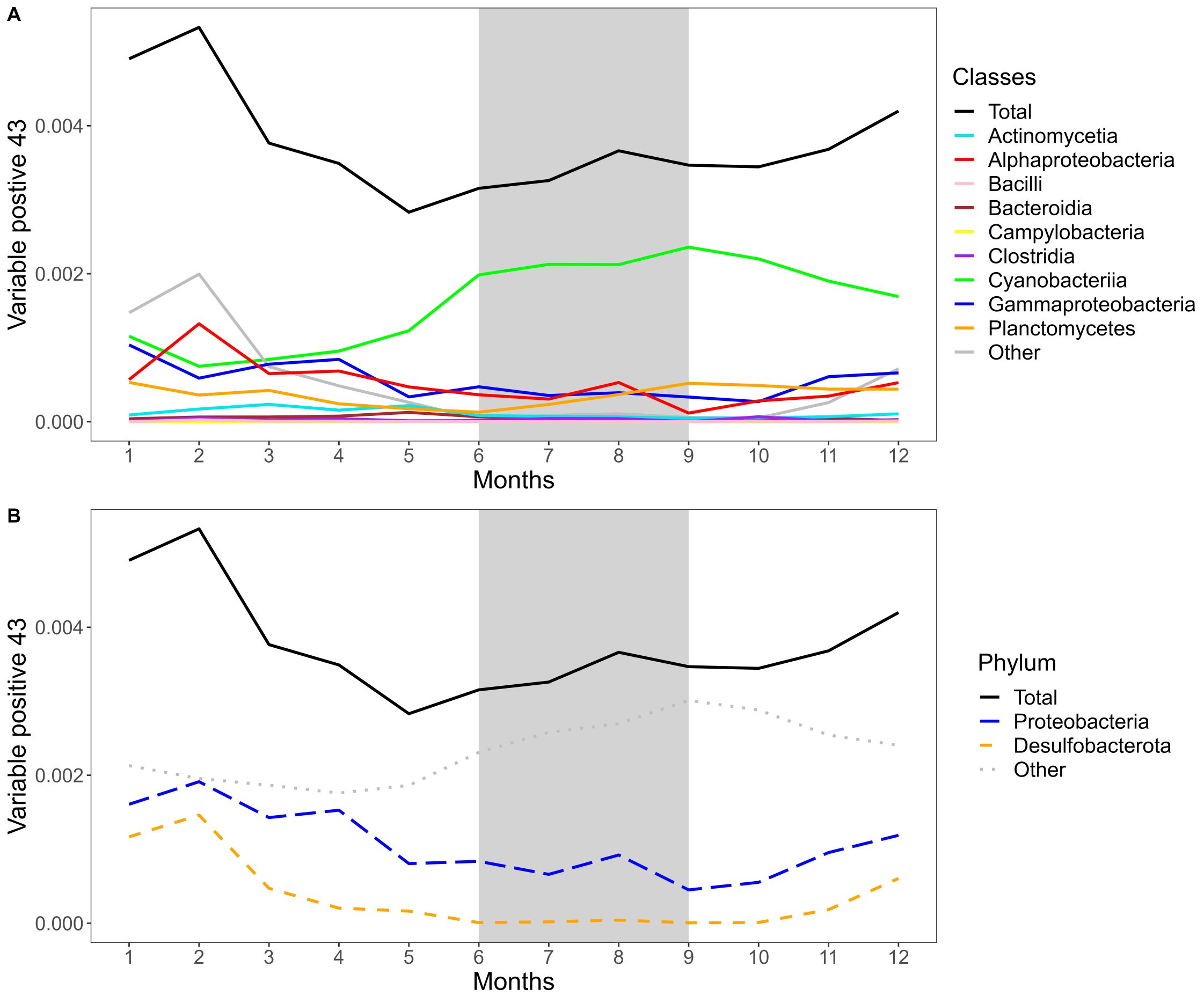

Supplement: FIG S6 — Abundance-weighted mean values of trait dominated by non-spore forming sulfate reducers over the yearly cycle. Summer months are indicated by a gray background. Taxonomic class is color-coded. [file msystems.00028-23-s0006.tif]
