# Supplementary material for: Quantification of metabolic niche occupancy dynamics in a Baltic Sea bacterial community
Source: mSystems. 2023 May 31;8(3):e00028-23. doi: 10.1128/msystems.00028-23 (PMC10312292; doi:10.1128/msystems.00028-23)
Supplement: TABLE S1 — Genomes that map to the 100 most abundant ASVs obtained from amplicon sequencing data in terms of relative mean abundance over the whole sampling period. Genome, taxonomic information, mean and maximum abundance over the whole sampling period are provided. [file msystems.00028-23-s0007.pdf]

Genome	Class	Family	Species	Mean abundance	Max abundance
GCF_003011885.1	Cyanobacteriia	Cyanobiaceae	Cyanobium_A usitatum	0.1150	0.7222
GCF_002252665.1	Cyanobacteriia	Cyanobiaceae	Cyanobium_A sp002252665	0.0533	0.2779
GCA_003569125.1	Acidimicrobiia	Ilumatobacteraceae	BACL27 sp003569125	0.0488	0.3656
GCA_001593825.1	Cyanobacteriia	Nostocaceae	Aphanizomenon_B flosaquae	0.0400	0.5437
GCF_000173115.1	Bacteroidia	Flavobacteriaceae	MAG-120531 sp000173115	0.0397	0.5443
GCA_002358295.1	Gammaproteobacteria	D2472	D2472 sp002358345	0.0287	0.2754
GCA_003569145.1	Actinomycetia	Nanopelagicaceae	MAG-120802 sp003569145	0.0286	0.2757
GCA_001438235.1	Alphaproteobacteria	Rhodobacteraceae	UBA10365 sp003536295	0.0235	0.1846
GCA_002405515.1	Planctomycetes	UBA1268	UBA4655 sp002405515	0.0226	0.1789
GCA_007280255.1	Planctomycetes	UBA1268	QWOQ01 sp003669585	0.0213	0.2010
GCA_001437765.1	Acidimicrobiia	Ilumatobacteraceae	UBA3006 sp002367695	0.0208	0.2445
GCA_002325485.1	Bacteroidia	Flavobacteriaceae	BACL21 sp002694465	0.0201	0.3039
GCA_002340585.1	Gammaproteobacteria	Porticoccaceae	HTCC2207 sp001438605	0.0191	0.2454
GCF_001485105.1	Actinomycetia	Streptomycetaceae	Streptomyces acidiscabies	0.0163	0.3385
GCA_002711735.1	Acidimicrobiia	Ilumatobacteraceae	Ilumatobacter_A sp002711735	0.0142	0.2166
GCF_000496475.1	Gammaproteobacteria	Burkholderiaceae	RS62 sp000496475	0.0139	0.1293
GCF_000257665.1	Actinomycetia	Microbacteriaceae	Aquiluna sp000257665	0.0129	0.2494
GCF_000312705.1	Cyanobacteriia	Nostocaceae	LE011-02 sp000312705	0.0122	0.5288
GCF_002287885.2	Actinomycetia	Nanopelagicaceae	Nanopelagicus limnes	0.0114	0.0733
GCF_000242915.1	Campylobacteria	Sulfurimonadaceae	Sulfurimonas gotlandica	0.0107	0.6154
GCA_002746305.1	Bacteroidia	UBA9320	UBA9320 sp002746305	0.0100	0.0928
GCA_002430225.1	Actinomycetia	Microbacteriaceae	Pontimonas sp001438965	0.0093	0.2095
GCF_002252705.1	Cyanobacteriia	Cyanobiaceae	Vulcanococcus limneticus	0.0092	0.1075
GCA_000750175.1	Alphaproteobacteria	Pelagibacteraceae	IMCC9063 sp000750175	0.0090	0.1093
GCA_002340845.1	Gammaproteobacteria	Methylophilaceae	BACL14 sp002384685	0.0079	0.0679
GCA_001438645.1	Gammaproteobacteria	Methylophilaceae	BACL14 sp002384685	0.0076	0.0700
GCA_001438145.1	Gammaproteobacteria	Pseudohongiellaceae	OM182 sp001438145	0.0074	0.0646
GCF_002284895.1	Actinomycetia	Nanopelagicaceae	Planktophila sp002284895	0.0074	0.0575
GCA_000485495.1	Actinomycetia	Nanopelagicaceae	AAA044-D11 sp000485495	0.0074	0.0642
GCA_002170165.1	Bacteroidia	BACL11	TMED123 sp002170165	0.0072	0.0501
GCF_900129545.1	Bacteroidia	Flavobacteriaceae	Flavobacterium fluvii	0.0060	0.2432
GCF_001983935.1	Planctomycetes	Planctomycetaceae	Fuerstia marisgermanicae	0.0058	0.1676
GCA_001438305.1	Bacteroidia	Schleiferiaceae	TMED14 sp001438205	0.0056	0.0693
GCF_002252635.1	Cyanobacteriia	Cyanobiaceae	WH-5701 sp002252635	0.0055	0.0868
GCA_004292795.1	Bacteroidia	Microscillaceae	RDXI01 sp004292795	0.0054	0.1049
GCF_006491595.1	Bacteroidia	Flavobacteriaceae	Flavobacterium jejuense	0.0052	0.0682
GCF_002943715.1	Bacteroidia	Flavobacteriaceae	Polaribacter filamentus	0.0052	0.1530
GCF_000299115.1	Alphaproteobacteria	HIMB59	HIMB59 sp000299115	0.0051	0.0616
GCF_900114485.1	Alphaproteobacteria	Rhodobacteraceae	Loktanella salsilacus	0.0049	0.0452
GCA_004379135.1	Acidimicrobiia	Ilumatobacteraceae	Casp-actino8 sp004379135	0.0048	0.0220
GCA_003249095.1	Cyanobacteriia	Microcystaceae	Snowella sp003249095	0.0047	0.1042
GCF_002631185.1	Alphaproteobacteria	Acetobacteraceae	Roseomonas rhizosphaerae	0.0047	0.3294
GCF_000738435.1	Alphaproteobacteria	Rhodobacteraceae	Planktomarina temperata	0.0046	0.1321
GCF_003096315.1	Gammaproteobacteria	Burkholderiaceae	Achromobacter insuavis	0.0046	0.4719
GCA_003284275.1	Alphaproteobacteria	Pelagibacteraceae	Pelagibacter_A sp003284275	0.0045	0.0547
GCF_002252675.1	Cyanobacteriia	Cyanobiaceae	Cyanobium sp002252675	0.0044	0.0434
GCF_002954645.1	Bacteroidia	Flavobacteriaceae	Polaribacter gangjinensis	0.0043	0.0744
GCF_002101315.1	Alphaproteobacteria	Pelagibacteraceae	Pelagibacter sp002101315	0.0040	0.0398
GCF_002115755.1	Alphaproteobacteria	Thalassospiraceae	Thalassospira mesophila	0.0039	0.2429
GCA_002733565.1	Gammaproteobacteria	Psychromonadaceae	Moritella sp000170855	0.0039	0.0718
GCF_002288225.1	Actinomycetia	Nanopelagicaceae	Planktophila dulcis	0.0039	0.0286
GCA_002428815.1	Gammaproteobacteria	Porticoccaceae	HTCC2207 sp001438605	0.0038	0.0499
GCF_000590925.1	Alphaproteobacteria	Rhodobacteraceae	Roseicyclus elongatum	0.0037	0.0555
GCA_002346275.1	Gammaproteobacteria	Halieaceae	IMCC3088 sp003520285	0.0037	0.0802
GCF_002940745.1	Bacteroidia	Flavobacteriaceae	Hanstruepera crassostreae	0.0036	0.0623
GCF_003335085.1	Bacteroidia	Flavobacteriaceae	Polaribacter sp003335085	0.0036	0.1667
GCF_000699505.1	Actinomycetia	Microbacteriaceae	Rhodoluna lacicola	0.0035	0.0265
GCA_003149555.1	Actinomycetia	Microbacteriaceae	Aquiluna sp003149555	0.0034	0.0827
GCA_004379115.1	Actinomycetia	S36-B12	Mxb001 sp004379115	0.0034	0.0716
GCA_001438005.1	Verrucomicrobiae	UBA3015	UBA3015 sp001438005	0.0032	0.0291
GCA_002346225.1	Bacteroidia	BACL12	UBA11426 sp002346225	0.0031	0.1118
GCF_003003055.1	Gammaproteobacteria	Burkholderiaceae	SCGC-AAA027-K21 sp003003055	0.0031	0.0317
GCF_002284855.1	Actinomycetia	Nanopelagicaceae	Planktophila sp002284855	0.0029	0.0379
GCA_002863125.1	Bacteroidia	UA16	UA16 sp002863125	0.0029	0.0250
GCF_002284915.1	Actinomycetia	Nanopelagicaceae	IMCC26077 sp002284915	0.0029	0.0279
GCA_001438165.1	Bacteroidia	Schleiferiaceae	TMED14 sp002381225	0.0029	0.0274
GCF_001457835.1	Clostridia	Ezakiellaceae	Fenollaria timonensis	0.0029	0.1789

GCF_000152785.1	Alphaproteobacteria	Rhodobacteraceae	Yoonia vestfoldensis_A	0.0028	0.0290
GCA_002292365.1	Bacteroidia	Cyclobacteriaceae	UBA4465 sp002292365	0.0028	0.0194
GCF_001439695.1	Gammaproteobacteria	Pseudomonadaceae	Pseudomonas_E veronii	0.0028	0.1510
GCA_000421325.1	Alphaproteobacteria	AAA536-G10	AAA536-G10 sp000421325	0.0027	0.0406
GCA_003208775.1	Cyanobacteriia	Cyanobiaceae	Synechococcus_C sp002500205	0.0026	0.1927
GCA_003671255.1	Planctomycetes	Gemmataceae	UBA969 sp003671255	0.0025	0.0379
GCA_007093895.1	Gammaproteobacteria	Enterobacteriaceae	Salmonella enterica	0.0024	0.0168
GCF_003011125.1	Cyanobacteriia	Cyanobiaceae	Synechococcus_D lacustris	0.0024	0.0380
GCF_004337435.1	Actinomycetia	Streptomycetaceae	Streptomyces sp004337435	0.0024	0.0120
GCA_002167745.1	Gammaproteobacteria	SG8-40	UBA3031 sp002167745	0.0023	0.0320
GCA_900618205.1	Gammaproteobacteria	Burkholderiaceae	Bordetella trematum	0.0023	0.1770
GCF_000173095.1	Bacteroidia	Flavobacteriaceae	MS024-2A sp000173095	0.0023	0.0428
GCF_000797465.1	Bacteroidia	Flavobacteriaceae	Psychroserpens jangbogonensis	0.0021	0.0377
GCA_002690755.1	Phycisphaerae	SM1A02	UBA12014 sp002690755	0.0021	0.0255
GCA_002480055.1	Gammaproteobacteria	Porticoccaceae	HTCC2207 sp002335945	0.0020	0.0215
GCF_000143825.1	Actinomycetia	Mycobacteriaceae	Corynebacterium genitalium_A	0.0020	0.0926
GCF_006385135.1	Alphaproteobacteria	Emcibacteraceae	Emcibacter_A congregatus	0.0020	0.0176
GCF_002284875.1	Actinomycetia	Nanopelagicaceae	Planktophila sp002284875	0.0019	0.0111
GCA_002697205.1	Gammaproteobacteria	HTCC2089	GCA-2697205 sp002697205	0.0019	0.0172
GCA_003045825.1	Bacteroidia	Schleiferiaceae	UBA10364 sp003045825	0.0019	0.0476
GCA_000762985.1	Actinomycetia	Mycobacteriaceae	Mycobacterium rufum	0.0018	0.0245
GCA_002282055.1	Bacteroidia	Sphingobacteriaceae	Daejeonella sp002257025	0.0018	0.0238
GCA_002499015.1	Poseidoniia	Poseidoniaceae	MGIIa-L1 sp002499015	0.0018	0.0959
GCF_000176015.1	Alphaproteobacteria	Rhodobacteraceae	Pseudorhodobacter_B sp000176015	0.0018	0.0476
GCF_006937785.1	Cyanobacteriia	Pseudanabaenaceae	Pseudanabaena sp006937785	0.0017	0.1092
GCF_001623485.1	Cyanobacteriia	Nostocaceae	Nodularia spumigena	0.0016	0.0348
GCF_000171835.1	Alphaproteobacteria	Thalassobaculaceae	BAL199 sp000171835	0.0015	0.0138
GCF_000156155.1	Gammaproteobacteria	Methylophilaceae	BACL14 sp000156155	0.0015	0.0159
GCA_002733945.1	Campylobacteria	Sulfurimonadaceae	Sulfurimonas sp002733945	0.0015	0.0763
GCF_003856375.1	Bacteroidia	Crocinitomicaceae	Fluviicola sp003856375	0.0015	0.0449
GCF_900110395.1	Alphaproteobacteria	Reyranellaceae	Reyranella sp900110395	0.0015	0.0222
GCF_002368115.1	Cyanobacteriia	Nostocaceae	Dolichospermum_A compactum	0.0014	0.0579
GCF_900100865.1	Actinomycetia	Microbacteriaceae	Aquiluna sp900100865	0.0014	0.0188
