# Supplementary material for: Quantification of metabolic niche occupancy dynamics in a Baltic Sea bacterial community
Source: mSystems. 2023 May 31;8(3):e00028-23. doi: 10.1128/msystems.00028-23 (PMC10312292; doi:10.1128/msystems.00028-23)
Supplement: TABLE S2 — Species that map to the 100 ASVs scoring most negative values in variable 1. [file msystems.00028-23-s0008.pdf]

Species	Value	Genome	Class	Family
Salmonella enterica	-0.09769	GCA_007094035.1	Gammaproteobacteria	Enterobacteriaceae
Salmonella enterica	-0.09769	GCA_007093895.1	Gammaproteobacteria	Enterobacteriaceae
Salmonella enterica	-0.09763	GCA_007093765.1	Gammaproteobacteria	Enterobacteriaceae
Salmonella enterica	-0.09756	GCF_003548795.1	Gammaproteobacteria	Enterobacteriaceae
Cronobacter sakazakii	-0.09754	GCF_002094495.1	Gammaproteobacteria	Enterobacteriaceae
Cronobacter sakazakii	-0.09754	GCF_002094665.1	Gammaproteobacteria	Enterobacteriaceae
Cronobacter sakazakii	-0.09754	GCF_002977865.1	Gammaproteobacteria	Enterobacteriaceae
Cronobacter turicensis	-0.09754	GCF_002976545.1	Gammaproteobacteria	Enterobacteriaceae
Cronobacter sakazakii	-0.09753	GCA_002094675.1	Gammaproteobacteria	Enterobacteriaceae
Cronobacter sakazakii	-0.09753	GCF_002094645.1	Gammaproteobacteria	Enterobacteriaceae
Cronobacter sakazakii	-0.09752	GCF_002977315.1	Gammaproteobacteria	Enterobacteriaceae
Cronobacter malonaticus	-0.09752	GCF_002978245.1	Gammaproteobacteria	Enterobacteriaceae
Cronobacter malonaticus	-0.09752	GCF_002978235.1	Gammaproteobacteria	Enterobacteriaceae
Cronobacter sakazakii	-0.09751	GCF_002094575.1	Gammaproteobacteria	Enterobacteriaceae
Cronobacter sakazakii	-0.09751	GCF_002976775.1	Gammaproteobacteria	Enterobacteriaceae
Cronobacter malonaticus	-0.09751	GCF_002978375.1	Gammaproteobacteria	Enterobacteriaceae
Cronobacter sakazakii	-0.09751	GCF_002094585.1	Gammaproteobacteria	Enterobacteriaceae
Cronobacter sakazakii	-0.09751	GCA_002976965.2	Gammaproteobacteria	Enterobacteriaceae
Cronobacter dublinensis	-0.09751	GCF_002979155.1	Gammaproteobacteria	Enterobacteriaceae
Cronobacter malonaticus	-0.09751	GCF_002978545.1	Gammaproteobacteria	Enterobacteriaceae
Cronobacter malonaticus	-0.09751	GCF_002978185.1	Gammaproteobacteria	Enterobacteriaceae
Cronobacter sakazakii	-0.09751	GCF_002977405.1	Gammaproteobacteria	Enterobacteriaceae
Cronobacter sakazakii	-0.09751	GCF_002094475.1	Gammaproteobacteria	Enterobacteriaceae
Cronobacter malonaticus	-0.0975	GCF_002978535.1	Gammaproteobacteria	Enterobacteriaceae
Cronobacter sakazakii	-0.0975	GCF_002976735.1	Gammaproteobacteria	Enterobacteriaceae
Cronobacter sakazakii	-0.0975	GCF_002976795.1	Gammaproteobacteria	Enterobacteriaceae
Cronobacter sakazakii	-0.0975	GCA_002977005.2	Gammaproteobacteria	Enterobacteriaceae
Escherichia coli	-0.09749	GCA_002078275.1	Gammaproteobacteria	Enterobacteriaceae
Cronobacter sakazakii	-0.09749	GCF_002977155.1	Gammaproteobacteria	Enterobacteriaceae
Escherichia coli	-0.09749	GCF_001268585.1	Gammaproteobacteria	Enterobacteriaceae
Escherichia coli	-0.09749	GCF_001269185.1	Gammaproteobacteria	Enterobacteriaceae
Escherichia coli	-0.09748	GCF_004523105.1	Gammaproteobacteria	Enterobacteriaceae
Escherichia coli	-0.09748	GCF_005889645.1	Gammaproteobacteria	Enterobacteriaceae
Escherichia coli	-0.09748	GCF_001268685.1	Gammaproteobacteria	Enterobacteriaceae
Escherichia coli	-0.09748	GCF_002007165.1	Gammaproteobacteria	Enterobacteriaceae
Escherichia coli	-0.09748	GCF_002959275.1	Gammaproteobacteria	Enterobacteriaceae
Cronobacter sakazakii	-0.09748	GCF_002978035.1	Gammaproteobacteria	Enterobacteriaceae
Cronobacter sakazakii	-0.09747	GCF_002978105.1	Gammaproteobacteria	Enterobacteriaceae
Cronobacter dublinensis	-0.09747	GCA_002978875.2	Gammaproteobacteria	Enterobacteriaceae
Cronobacter dublinensis	-0.09747	GCF_002978655.1	Gammaproteobacteria	Enterobacteriaceae
Enterobacter sp.	-0.09746	GCF_000534395.1	Gammaproteobacteria	Enterobacteriaceae
Scandinavium goeteborgense	-0.09744	GCF_004361715.1	Gammaproteobacteria	Enterobacteriaceae
Scandinavium goeteborgense	-0.09743	GCA_003935895.2	Gammaproteobacteria	Enterobacteriaceae
Klebsiella pneumoniae	-0.09742	GCF_003967395.1	Gammaproteobacteria	Enterobacteriaceae
Cronobacter dublinensis	-0.09742	GCF_002978855.1	Gammaproteobacteria	Enterobacteriaceae
Klebsiella quasivariicola	-0.09742	GCF_002269255.1	Gammaproteobacteria	Enterobacteriaceae
Klebsiella variicola	-0.09742	GCF_001033575.1	Gammaproteobacteria	Enterobacteriaceae
Klebsiella quasipneumoniae	-0.09742	GCF_002853635.1	Gammaproteobacteria	Enterobacteriaceae
Klebsiella pneumoniae	-0.09742	GCF_004127885.1	Gammaproteobacteria	Enterobacteriaceae
Citrobacter freundii	-0.09741	GCA_001686345.1	Gammaproteobacteria	Enterobacteriaceae
Enterobacter cloacae	-0.09741	GCF_001562175.1	Gammaproteobacteria	Enterobacteriaceae
Enterobacter sp.	-0.0974	GCF_000493015.1	Gammaproteobacteria	Enterobacteriaceae
Cronobacter sakazakii	-0.0974	GCF_002977115.1	Gammaproteobacteria	Enterobacteriaceae
Citrobacter koseri	-0.09739	GCF_002393245.1	Gammaproteobacteria	Enterobacteriaceae
Klebsiella pneumoniae	-0.09739	GCF_003227185.1	Gammaproteobacteria	Enterobacteriaceae
Klebsiella pneumoniae	-0.09739	GCF_004127515.1	Gammaproteobacteria	Enterobacteriaceae
Pantoea sp.	-0.09737	GCF_002920175.1	Gammaproteobacteria	Enterobacteriaceae
Klebsiella grimontii	-0.09736	GCA_902159485.1	Gammaproteobacteria	Enterobacteriaceae
Enterobacter sp.	-0.09736	GCA_007035975.1	Gammaproteobacteria	Enterobacteriaceae
Pantoea sp.	-0.09736	GCF_002313185.2	Gammaproteobacteria	Enterobacteriaceae
Pantoea sp.	-0.09736	GCF_003813865.1	Gammaproteobacteria	Enterobacteriaceae
Buttiauxella izardii	-0.09735	GCF_003601925.1	Gammaproteobacteria	Enterobacteriaceae
Klebsiella grimontii	-0.09735	GCA_902158675.1	Gammaproteobacteria	Enterobacteriaceae
Lelliottia nimipressuralis	-0.09734	GCF_004402045.1	Gammaproteobacteria	Enterobacteriaceae
Pantoea stewartii	-0.09732	GCF_001310295.1	Gammaproteobacteria	Enterobacteriaceae
Pantoea sp.	-0.09729	GCF_000963985.1	Gammaproteobacteria	Enterobacteriaceae
Rahnella aquatilis	-0.09729	GCF_000735505.1	Gammaproteobacteria	Enterobacteriaceae

Buttiauxella sp.	-0.09728	GCF_003675305.1	Gammaproteobacteria	Enterobacteriaceae
Cronobacter dublinensis	-0.09727	GCF_002978705.2	Gammaproteobacteria	Enterobacteriaceae
Erwinia sp.	-0.09726	GCF_002752575.1	Gammaproteobacteria	Enterobacteriaceae
Rouxiella chamberiensis	-0.09724	GCF_000951135.1	Gammaproteobacteria	Enterobacteriaceae
Erwinia sp.	-0.09724	GCF_004551645.1	Gammaproteobacteria	Enterobacteriaceae
Enterobacter sp.	-0.09721	GCF_000277545.1	Gammaproteobacteria	Enterobacteriaceae
Gamma proteobacterium	-0.09715	GCA_000335795.1	Gammaproteobacteria	Enterobacteriaceae
Rahnella aquatilis	-0.09711	GCA_003956145.2	Gammaproteobacteria	Enterobacteriaceae
Rahnella woolbedingensis	-0.09709	GCF_003602095.1	Gammaproteobacteria	Enterobacteriaceae
Ewingella americana	-0.09702	GCF_006438725.1	Gammaproteobacteria	Enterobacteriaceae
Ewingella americana	-0.09696	GCF_900451015.1	Gammaproteobacteria	Enterobacteriaceae
Serratia quinivorans	-0.09689	GCF_900457075.1	Gammaproteobacteria	Enterobacteriaceae
Atlantibacter hermannii	-0.09685	GCF_900635495.1	Gammaproteobacteria	Enterobacteriaceae
Serratia proteamaculans	-0.09685	GCF_004684015.1	Gammaproteobacteria	Enterobacteriaceae
Serratia sp.	-0.09684	GCF_002607755.1	Gammaproteobacteria	Enterobacteriaceae
Pseudescherichia vulneris	-0.09681	GCF_900450975.1	Gammaproteobacteria	Enterobacteriaceae
Buttiauxella sp.	-0.09678	GCF_006376615.1	Gammaproteobacteria	Enterobacteriaceae
Pantoea vagans	-0.09644	GCF_001506165.1	Gammaproteobacteria	Enterobacteriaceae
Ewingella americana	-0.09632	GCF_000735345.1	Gammaproteobacteria	Enterobacteriaceae
Pectobacterium carotovorum	-0.096	GCF_002250215.1	Gammaproteobacteria	Enterobacteriaceae
Serratia fonticola	-0.09576	GCF_006714955.1	Gammaproteobacteria	Enterobacteriaceae
Serratia sp.	-0.0957	GCF_003668775.1	Gammaproteobacteria	Enterobacteriaceae
Yersinia enterocolitica	-0.09544	GCF_002082245.2	Gammaproteobacteria	Enterobacteriaceae
Yersinia enterocolitica	-0.09536	GCF_002083285.2	Gammaproteobacteria	Enterobacteriaceae
Yersinia kristensenii	-0.09526	GCF_002188895.1	Gammaproteobacteria	Enterobacteriaceae
Morganella morganii	-0.05621	GCF_003287815.1	Gammaproteobacteria	Enterobacteriaceae
Plesiomonas sp.	-0.01279	GCF_000800945.1	Gammaproteobacteria	Enterobacteriaceae
Plesiomonas shigelloides	-0.01279	GCF_002093895.1	Gammaproteobacteria	Enterobacteriaceae
Aeromonas jandaei	-0.01248	GCF_000708125.1	Gammaproteobacteria	Aeromonadaceae
Aeromonas veronii	-0.01245	GCF_000298015.1	Gammaproteobacteria	Aeromonadaceae
Photobacterium kishitanii	-0.01235	GCF_003025945.1	Gammaproteobacteria	Vibrionaceae
Photobacterium phosphoreum	-0.01235	GCF_003025815.1	Gammaproteobacteria	Vibrionaceae
Aeromonas popoffii	-0.01224	GCF_000820025.1	Gammaproteobacteria	Aeromonadaceae
