# Supplementary material for: Quantification of metabolic niche occupancy dynamics in a Baltic Sea bacterial community
Source: mSystems. 2023 May 31;8(3):e00028-23. doi: 10.1128/msystems.00028-23 (PMC10312292; doi:10.1128/msystems.00028-23)
Supplement: TABLE S3 — Top 100 over-represented annotated genes in the genomes of the taxa that receive the most negative entries on variable 1 negative (A), variable 2 positive (B), variable 3 positive (C), variable 4 negative (D), variable 14 negative (E), variable 38 negative (F), variable 43 positive (G). The NES and FDR-Adj. P columns show the normalized ’Enrichment score’ and FDR-adjusted (43) P-value from the enrichment analysis (40). [file msystems.00028-23-s0009.pdf]

A

**Gene**	**FDR-Adj. P**	**NES**
Major outer membrane lipoprotein Lpp 1	-11.844	0.00025
Phage shock protein G	-11.798	0.00025
Primosomal replication protein N”	-11.753	0.00025
DNA damage-inducible protein I	-11.694	0.00025
Outer membrane porin C	-11.614	0.00025
Chaperone protein YcdY	-11.562	0.00025
USG-1 protein	-11.506	0.00025
HTH-type transcriptional regulator cbl	-11.357	0.00025
Inner membrane protein YghB	-11.34	0.00025
Cytochrome c-type protein NrfB	-11.321	0.00025
Plasmid partition protein A	-11.288	0.00025
Protein rof	-11.241	0.00025
putative inner membrane protein Smp	-11.208	0.00025
putative protein YbjN	-11.199	0.00025
putative lipoprotein YbaY	-11.157	0.00025
putative HTH-type transcriptional regulator YbdO	-11.112	0.00025
Inner membrane protein YqjE	-11.063	0.00025
putative protein YfeY	-11.051	0.00025
Secretion monitor	-11.041	0.00025
putative ECA polymerase	-11.035	0.00025
Protein Sxy	-10.994	0.00025
Kdo(2)-lipid A phosphoethanolamine 7”-transferase	-10.986	0.00025
Intermembrane phospholipid transport system binding protein MlaB	-10.98	0.00025
putative cyclic di-GMP phosphodiesterase PdeD	-10.94	0.00025
Phosphatidylglycerophosphatase C	-10.93	0.00025
Inner membrane protein YlaC	-10.922	0.00025
Multiple stress resistance protein BhsA	-10.922	0.00025
Protein YdgH	-10.922	0.00025
Protein PhoH	-10.898	0.00025
Outer membrane protein X	-10.882	0.00025
Sensor protein BasS	-10.881	0.00025
Inner membrane protein YgbE	-10.872	0.00025
putative protein YjjI	-10.872	0.00025
Biofilm regulator BssS	-10.87	0.00025
DNA polymerase III subunit theta	-10.87	0.00025
Multidrug efflux pump accessory protein AcrZ	-10.87	0.00025
putative protein YejG	-10.87	0.00025
putative protein YhcO	-10.87	0.00025
Protein YhjJ	-10.864	0.00025
Constitutive lysine decarboxylase	-10.863	0.00025
Pirin-like protein YhaK	-10.861	0.00025

Constitutive ornithine decarboxylase	-10.843	0.00025
Inner membrane protein YdgK	-10.817	0.00025
putative cyclic di-GMP phosphodiesterase PdeK	-10.815	0.00025
Flagellar regulator flk	-10.81	0.00025
Lipoprotein BsmA	-10.81	0.00025
Modulator protein MzrA	-10.81	0.00025
Inner membrane protein YgfX	-10.808	0.00025
Protein DsrB	-10.808	0.00025
Phosphoethanolamine transferase OpgE	-10.764	0.00025
Transcriptional regulatory protein RcsA	-10.748	0.00025
Osmotically-inducible lipoprotein B	-10.748	0.00025
Cyclic di-GMP phosphodiesterase PdeH	-10.735	0.00025
Hha toxicity modulator TomB	-10.732	0.00025
Type II secretion system protein H	-10.728	0.00025
putative lipoprotein YajI	-10.69	0.00025
putative protein YebV	-10.682	0.00025
Inner membrane protein YbjO	-10.682	0.00025
putative lipoprotein YbjP	-10.682	0.00025
putative protein YccJ	-10.663	0.00025
Inner membrane protein YfeZ	-10.655	0.00025
Flagella synthesis protein FlgN	-10.649	0.00025
Periplasmic chaperone Spy	-10.637	0.00025
putative ferredoxin-like protein YdhX	-10.629	0.00025
Regulatory protein SoxS	-10.485	0.00025
Protein TonB	-10.47	0.00025
Cyclic di-GMP binding protein BcsE	-10.384	0.00025
Quorum-sensing regulator protein G	-10.381	0.00025
Signal transduction histidine-protein kinase/phosphatase UhpB	-10.368	0.00025
Regulator of sigma S factor FliZ	-10.328	0.00025
Inner membrane protein YbjM	-10.323	0.00025
Trimethylamine-N-oxide reductase	-10.318	0.00025
Ferric iron reductase protein FhuF	-10.31	0.00025
putative protein YaiA	-10.29	0.00025
Negative regulator of flagellin synthesis	-10.282	0.00025
Anti-adapter protein IraP	-10.27	0.00025
Ferric enterobactin transport protein FepE	-10.235	0.00025
Intracellular growth attenuator protein igaA	-10.225	0.00025
Outer membrane porin N	-10.133	0.00025
Flagellar protein FlhE	-10.095	0.00025
Inner membrane protein YebE	-10.087	0.00025
HTH-type transcriptional repressor BluR	-10.05	0.00025
Enterobactin synthase component F	-10.035	0.00025
Sec-independent protein translocase protein TatE	-9.992	0.00025

Alternative ribosome-rescue factor A	-9.987	0.00025
Primosomal protein 1	-9.919	0.00025
putative csgAB operon transcriptional regulatory protein	-9.909	0.00025
putative protein YgaM	-9.887	0.00025
Multiple antibiotic resistance protein MarA	-9.846	0.00025
Inner membrane protein YbhQ	-9.832	0.00025
Inner membrane protein YjiG	-9.829	0.00025
Putative selenoprotein YdfZ	-9.827	0.00025
HTH-type transcriptional regulator MlrA	-9.802	0.00025
Tryptophanase	-9.8	0.00025
Universal stress protein C	-9.798	0.00025
Hemolysin expression-modulating protein Hha	-9.771	0.00025
6-phospho-beta-glucosidase BglB	-9.762	0.00025
Ribonucleoside-diphosphate reductase 2 subunit alpha	-9.711	0.00025
Transcriptional regulator SutA	-9.71	0.00025
Cyclic di-GMP binding protein	-9.705	0.00025

B

**Gene**	**FDR-Adj. P**	**NES**
Outer-membrane lipoprotein LolB	15.094	0.0004
DNA-binding protein Fis	15.02	0.0004
LPS-assembly lipoprotein LptE	15.004	0.0004
Ammonia monooxygenase gamma subunit	14.873	0.0004
Thiol:disulfide interchange protein DsbA	14.848	0.0004
DNA polymerase III subunit delta	14.719	0.0004
Phosphate regulon sensor protein PhoR	14.697	0.0004
Pyruvate kinase II	14.344	0.0004
1,6-anhydro-N-acetylmuramyl-L-alanine amidase AmpD	14.232	0.0004
Cell division protein FtsN	14.223	0.0004
Stringent starvation protein A	14.185	0.0004
Lipopolysaccharide export system permease protein LptF	14.002	0.0004
Succinate dehydrogenase hydrophobic membrane anchor subunit	13.978	0.0004
Recombination-associated protein RdgC	13.944	0.0004
Modulator of FtsH protease YccA	13.94	0.0004
Cell division protein ZipA	13.785	0.0004
Cbb3-type cytochrome c oxidase subunit CcoN1	13.784	0.0004
Cytochrome c4	13.777	0.0004
2-octaprenylphenol hydroxylase	13.656	0.0004
2-octaprenyl-6-methoxyphenol hydroxylase	13.643	0.0004
Phosphoenolpyruvate synthase regulatory protein	13.401	0.0004
HTH-type transcriptional regulator CysB	13.346	0.0004
2-methyl-aconitate isomerase	13.193	0.0004
Ferredoxin 1	13.12	0.0004

Intermembrane phospholipid transport system ATP-binding protein MlaF	13.091	0.0004
Large ribosomal RNA subunit accumulation protein YceD	13.051	0.0004
Cytoskeleton protein RodZ	13.043	0.0004
Chorismate pyruvate-lyase	12.981	0.0004
Soluble lytic murein transglycosylase	12.981	0.0004
Intermembrane phospholipid transport system binding protein MlaD	12.951	0.0004
2Fe-2S ferredoxin	12.898	0.0004
Cell division protein DedD	12.872	0.0004
Protein YcgL	12.871	0.0004
Uroporphyrinogen-III synthase	12.748	0.0004
High frequency lysogenization protein HflD	12.712	0.0004
putative protein YaeQ	12.679	0.0004
Exodeoxyribonuclease I	12.653	0.0004
Co-chaperone protein HscB	12.641	0.0004
Phosphatase NudJ	12.606	0.0004
Glutamate-pyruvate aminotransferase AlaA	12.599	0.0004
Ribonuclease T	12.541	0.0004
Intermembrane phospholipid transport system binding protein MlaC	12.528	0.0004
Colicin V production protein	12.514	0.0004
5-amino-6-(5-phospho-D-ribitylamino)uracil phosphatase YigB	12.51	0.0004
Fimbrial protein	12.493	0.0004
Inner membrane transport protein YajR	12.469	0.0004
50S ribosomal protein L16 3-hydroxylase	12.462	0.0004
Protein phosphatase CheZ	12.411	0.0004
FKBP-type 16 kDa peptidyl-prolyl cis-trans isomerase	12.376	0.0004
putative protein YibN	12.342	0.0004
Molybdopterin-synthase adenylyltransferase	12.331	0.0004
Inner membrane protein YpjD	12.312	0.0004
Iron-sulfur cluster assembly protein CyaY	12.218	0.0004
Sigma factor AlgU regulatory protein MucB	12.217	0.0004
Protein-glutamine gamma-glutamyltransferase	12.205	0.0004
Pyrimidine/purine nucleotide 5’-monophosphate nucleosidase	12.141	0.0004
Bifunctional (p)ppGpp synthase/hydrolase SpoT	12.134	0.0004
Putative glutamine amidotransferase YafJ	12.126	0.0004
Penicillin-binding protein 1B	12.077	0.0004
CDP-6-deoxy-L-threo-D-glycero-4-hexulose-3-dehydrase reductase	12.042	0.0004
Peptidoglycan hydrolase FlgJ	12.011	0.0004
Methylmalonate-semialdehyde dehydrogenase [acylating]	11.998	0.0004
Acid stress protein IbaG	11.995	0.0004
Ribosome modulation factor	11.907	0.0004
Cbb3-type cytochrome c oxidase subunit CcoP2	11.904	0.0004
ATP synthase subunit beta 1	11.902	0.0004
3-deoxy-D-manno-octulosonic acid kinase	11.79	0.0004

ADP compounds hydrolase NudE	11.777	0.0004
HTH-type transcriptional regulator YhaJ	11.776	0.0004
Nucleoid-associated protein YejK	11.714	0.0004
Sensor protein QseC	11.713	0.0004
UTP pyrophosphatase	11.706	0.0004
ATP-dependent DNA helicase DinG	11.685	0.0004
Riboflavin transporter	11.672	0.0004
Membrane-bound lytic murein transglycosylase B	11.611	0.0004
putative acyltransferase YihG	11.598	0.0004
Protein ImuB	11.561	0.0004
HTH-type transcriptional regulator HmrR	11.528	0.0004
DNA polymerase III subunit chi	11.502	0.0004
Flagellar basal-body rod protein FlgF	11.498	0.0004
Outer membrane protein assembly factor BamC	11.498	0.0004
Aerotaxis receptor	11.478	0.0004
Protein-glutamate methylesterase/protein-glutamine glutaminase 1	11.468	0.0004
Dihydroorotase-like protein	11.451	0.0004
Protein YciI	11.451	0.0004
Cell division protein ZapD	11.45	0.0004
Peptidyl-prolyl cis-trans isomerase cyp18	11.442	0.0004
FAD assembly factor SdhE	11.418	0.0004
D-erythrose-4-phosphate dehydrogenase	11.393	0.0004
putative DNA endonuclease SmrA	11.37	0.0004
Protein Smg	11.35	0.0004
Type II secretion system protein K	11.347	0.0004
Cysteine synthase A	11.339	0.0004
Exoribonuclease 2	11.332	0.0004
Chaperone protein HscA	11.319	0.0004
tRNA/tmRNA (uracil-C(5))-methyltransferase	11.319	0.0004
Murein hydrolase activator NlpD	11.28	0.0004
Methyl-accepting chemotaxis protein McpP	11.26	0.0004
Glutathione S-transferase GST-6.0	11.243	0.0004
Methyl-accepting chemotaxis protein McpH	11.228	0.0004

C

**Gene**	**FDR-Adj. P**	**NES**
GTP pyrophosphokinase rsh	12.285	0.00054
putative peptidoglycan D,D-transpeptidase FtsI	12.172	0.00054
Chromosome-partitioning protein ParB	12.152	0.00054
5-aminolevulinate synthase	12.02	0.00054
FtsZ-localized protein C	11.923	0.00054
6,7-dimethyl-8-ribityllumazine synthase 1	11.812	0.00054
FtsZ-localized protein A	11.775	0.00054

Aerobic cobaltochelatase subunit CobT	11.742	0.00054
Phyllosphere-induced regulator PhyR	11.705	0.00054
NADH-quinone oxidoreductase chain 1	11.649	0.00054
Ubiquinone hydroxylase UbiL	11.525	0.00054
Cell cycle response regulator CtrA	11.398	0.00054
Protein phosphotransferase ChpT	11.394	0.00054
Heat shock protein HspQ	11.378	0.00054
Aerobic cobaltochelatase subunit CobS	11.303	0.00054
Ferredoxin-2	11.301	0.00054
Dihydrolipoyl dehydrogenase 3	11.181	0.00054
flagellum biosynthesis repressor protein FlbT	11.152	0.00054
Cytochrome c oxidase subunit 1 , bacteroid	11.079	0.00054
Thiol:disulfide interchange protein CycY	11.068	0.00054
putative protein RP812	10.703	0.00054
Polyphosphate:NDP phosphotransferase 3	10.652	0.00054
Serine hydroxymethyltransferase 2	10.633	0.00054
Transcriptional regulatory protein ros	10.535	0.00054
Ferredoxin-6	10.444	0.00054
Glutamate–cysteine ligase EgtA	10.444	0.00054
Propionyl-CoA carboxylase regulator	10.367	0.00054
RNA polymerase sigma-54 factor 2	10.354	0.00054
Bifunctional enzyme IspD/IspF	10.297	0.00054
Cytochrome c1	10.28	0.00054
Cold shock protein CspA	10.25	0.00054
Thiol:disulfide interchange protein TlpA	10.212	0.00054
Nitrogen fixation regulation protein FixK	10.079	0.00054
Cytochrome c oxidase subunit 1-beta	9.815	0.00054
NADH-quinone oxidoreductase chain 5	9.719	0.00054
(3S)-malyl-CoA thioesterase	9.564	0.00054
UDP-2,3-diacylglucosamine pyrophosphatase LpxI	9.371	0.00054
ATP synthase protein I	9.331	0.00054
Hypotaurine/taurine–pyruvate aminotransferase	9.33	0.00054
Blue-light-activated histidine kinase	9.329	0.00054
HTH-type transcriptional regulator RamB	9.327	0.00054
Porin	9.325	0.00054
Penicillin-insensitive murein endopeptidase	9.315	0.00054
Glycine betaine methyltransferase	9.294	0.00054
L-arabinose 1-dehydrogenase (NAD(P)(+))	9.267	0.00054
Putative metal-sulfur cluster biosynthesis proteins YuaD	9.264	0.00054
ATP synthase subunit b’	9.25	0.00054
N-acetylmuramoyl-L-alanine amidase AmiD	9.212	0.00054
Periplasmic alpha-galactoside-binding protein	9.195	0.00054
10 kDa chaperonin 1	9.186	0.00054

Urease subunit gamma 1	9.152	0.00054
(2S)-methylsuccinyl-CoA dehydrogenase	9.097	0.00054
Urease subunit alpha 1	9.066	0.00054
Hemolysin C	9.047	0.00054
Urease accessory protein UreE 1	8.894	0.00054
Lysine/ornithine decarboxylase	8.874	0.00054
Dicamba O-demethylase 1, ferredoxin reductase component	8.873	0.00054
Serine–glyoxylate aminotransferase	8.855	0.00054
Precorrin-3B C(17)-methyltransferase	8.849	0.00054
Nicotinate phosphoribosyltransferase	8.819	0.00054
Glycogen synthase 1	8.812	0.00054
3-hydroxybenzoate 6-hydroxylase 1	8.768	0.00054
nicotinate-nucleotide adenylyltransferase	8.741	0.00054
Molybdenum cofactor insertion chaperone PaoD	8.735	0.00054
Arginine–pyruvate transaminase AruH	8.718	0.00054
D-hydantoinase/dihydropyrimidinase	8.695	0.00054
putative 3-hydroxyisobutyrate dehydrogenase	8.592	0.00054
Response regulator receiver protein CpdR	8.56	0.00054
60 kDa chaperonin 5	8.547	0.00054
Lysophospholipase L2	8.545	0.00054
Type I secretion system ATP-binding protein PrsD	8.544	0.00054
Phosphatidylcholine synthase	8.531	0.00054
Carbonic anhydrase 1	8.507	0.00054
Bifunctional coenzyme PQQ synthesis protein C/D	8.503	0.00054
NAD-dependent dihydropyrimidine dehydrogenase subunit PreT	8.474	0.00054
Anti-sigma-F factor NrsF	8.456	0.00054
FAD-dependent catabolic D-arginine dehydrogenase DauA	8.437	0.00054
Nopaline-binding periplasmic protein	8.432	0.00054
Mesaconyl-CoA hydratase	8.431	0.00054
putative riboflavin import permease protein RfuD	8.419	0.00054
Sulfite dehydrogenase subunit C	8.391	0.00054
Crotonyl-CoA carboxylase/reductase	8.381	0.00054
Precorrin-2 C(20)-methyltransferase	8.38	0.00054
Acyl carrier protein AcpXL	8.376	0.00054
Polysialic acid transport ATP-binding protein KpsT	8.372	0.00054
Alkane 1-monooxygenase 2	8.352	0.00054
HTH-type transcriptional regulator RafR	8.331	0.00054
S-formylglutathione hydrolase	8.326	0.00054
Ethylmalonyl-CoA mutase	8.282	0.00054
Outer membrane protein	8.273	0.00054
Cytochrome c oxidase subunit 4	8.252	0.00054
Cytochrome c-556	8.237	0.00054
Alpha-D-ribose 1-methylphosphonate 5-triphosphate synthase subunit PhnG	8.236	0.00054

Alpha-D-ribose 1-methylphosphonate 5-triphosphate diphosphatase	8.223	0.00054
Sulfite dehydrogenase subunit A	8.218	0.00054
Glutathione-specific gamma-glutamylcyclotransferase	8.17	0.00054
(S)-ureidoglycine aminohydrolase	8.165	0.00054
Hydrogenobyrinate a,c-diamide synthase	8.101	0.00054
Alanine racemase, biosynthetic	8.068	0.00054
Alpha-D-ribose 1-methylphosphonate 5-triphosphate synthase subunit PhnH	8.045	0.00054

D

**Gene**	**FDR-Adj. P**	**NES**
Cytochrome f	-6.889	0.00031
Photosystem II manganese-stabilizing polypeptide	-6.876	0.00031
Protein Thf1	-6.874	0.00031
Photosystem II CP47 reaction center protein	-6.859	0.00031
NAD(P)H-quinone oxidoreductase subunit N	-6.857	0.00031
Photosystem I assembly protein Ycf4	-6.857	0.00031
Photosystem I reaction center subunit III	-6.857	0.00031
Phycocyanobilin:ferredoxin oxidoreductase	-6.85	0.00031
Photosystem II reaction center Psb28 protein	-6.836	0.00031
Photosystem II lipoprotein Psb27	-6.835	0.00031
NAD(P)H-quinone oxidoreductase subunit O	-6.833	0.00031
Photosystem I P700 chlorophyll a apoprotein A1	-6.833	0.00031
Photosystem II reaction center protein K	-6.829	0.00031
Cytochrome b559 subunit alpha	-6.826	0.00031
Photosystem II CP43 reaction center protein	-6.826	0.00031
Photosystem I reaction center subunit IV	-6.824	0.00031
30S ribosomal protein S21 A	-6.812	0.00031
Pentapeptide repeat protein Rfr32	-6.807	0.00031
Photosystem II reaction center protein H	-6.804	0.00031
Ycf54-like protein	-6.803	0.00031
Ferredoxin-thioredoxin reductase, catalytic chain	-6.802	0.00031
NAD(P)H-quinone oxidoreductase subunit M	-6.795	0.00031
Long-chain acyl-[acyl-carrier-protein] reductase	-6.795	0.00031
Bifunctional pantoate ligase/cytidylate kinase	-6.792	0.00031
RNA polymerase sigma factor SigA2	-6.78	0.00031
Photosystem I reaction center subunit II	-6.779	0.00031
Photosystem I reaction center subunit XI	-6.779	0.00031
Phycobiliprotein beta chain	-6.773	0.00031
Phycobilisome 7.8 kDa linker polypeptide, allophycocyanin-associated, core	-6.773	0.00031
Photosystem I reaction center subunit XII	-6.769	0.00031
Aldehyde decarbonylase	-6.752	0.00031
Photosystem II reaction center protein Z	-6.75	0.00031
Ferredoxin-thioredoxin reductase, variable chain	-6.739	0.00031

Photosystem II 12 kDa extrinsic protein	-6.73	0.00031
Photosystem II protein Y	-6.723	0.00031
Protein PsbN	-6.723	0.00031
Proton extrusion protein PcxA	-6.719	0.00031
Cytochrome b6-f complex subunit 7	-6.698	0.00031
Phycocyanobilin lyase subunit alpha	-6.692	0.00031
Orange carotenoid-binding protein	-6.684	0.00031
Cytochrome b559 subunit beta	-6.665	0.00031
Photosystem I iron-sulfur center	-6.638	0.00031
Vitamin K epoxide reductase	-6.628	0.00031
Photosystem I reaction center subunit IX	-6.626	0.00031
ATP-dependent zinc metalloprotease FtsH 2	-6.612	0.00031
Putative diflavin flavoprotein A 3	-6.577	0.00031
putative glutaredoxin	-6.556	0.00031
Monoglucosyldiacylglycerol epimerase	-6.546	0.00031
Photosystem II protein D1 2	-6.546	0.00031
NAD(P)H-quinone oxidoreductase subunit L	-6.543	0.00031
Lipoyl synthase 2	-6.517	0.00031
Phycocyanobilin lyase CpcT	-6.495	0.00031
2-methyl-6-phytyl-1,4-hydroquinone methyltransferase	-6.492	0.00031
Photosystem II reaction center X protein	-6.491	0.00031
Photosystem II D2 protein	-6.47	0.00031
Transcription regulator LexA	-6.463	0.00031
Ycf53-like protein	-6.463	0.00031
D-fructose 1,6-bisphosphatase class 2/sedoheptulose 1,7-bisphosphatase	-6.447	0.00031
putative arabinosyltransferase C	-6.445	0.00031
Phycobiliprotein ApcE	-6.435	0.00031
Photosystem II reaction center protein T	-6.434	0.00031
NAD(P)H-quinone oxidoreductase subunit K 1	-6.424	0.00031
Sensor protein SphS	-6.419	0.00031
Allophycocyanin beta chain	-6.412	0.00031
Photosystem II reaction center protein M	-6.396	0.00031
Photosystem II reaction center protein Ycf12	-6.39	0.00031
Circadian clock protein KaiA	-6.388	0.00031
Photosystem I reaction center subunit VIII	-6.371	0.00031
High-affinity Na(+)/H(+) antiporter NhaS3	-6.34	0.00031
putative 30S ribosomal protein PSRP-3	-6.308	0.00031
Isoaspartyl peptidase/L-asparaginase	-6.297	0.00031
Phytol kinase	-6.261	0.00031
Regulatory protein CysR	-6.248	0.00031
Photosystem II reaction center protein I	-6.243	0.00031
Serine/threonine-protein kinase B	-6.237	0.00031
Ferredoxin-dependent glutamate synthase 2	-6.229	0.00031

Phycobilisome rod-core linker polypeptide CpcG	-6.226	0.00031
Galactan 5-O-arabinofuranosyltransferase	-6.217	0.00031
NAD(P)H-quinone oxidoreductase subunit J	-6.213	0.00031
Putative serine protease HhoA	-6.174	0.00031
Phycocyanobilin lyase subunit CpcS	-6.151	0.00031
Photosystem II reaction center protein J	-6.149	0.00031
Putative acetyl-coenzyme A carboxylase carboxyl transferase subunit beta	-6.134	0.00031
Phycocyanobilin lyase subunit beta	-6.125	0.00031
NAD(P)H-quinone oxidoreductase subunit I	-6.123	0.00031
Phosphoribulokinase	-6.118	0.00031
Putative isochorismate synthase MenF	-6.103	0.00031
Allophycocyanin subunit alpha-B	-6.097	0.00031
Putative cytochrome P450 120	-6.075	0.00031
Putative diflavin flavoprotein A 5	-6.071	0.00031
Bicarbonate-binding protein CmpA	-6.071	0.00031
C-phycocyanin beta chain	-6.068	0.00031
Chromophore lyase CpcS/CpeS	-6.065	0.00031
4-hydroxybenzoate solanesyltransferase	-6.034	0.00031
Hydrolase	-6.024	0.00031
L,D-transpeptidase 2	-6.004	0.00031
putative ferredoxin/ferredoxin–NADP reductase	-5.991	0.00031
Photosystem II reaction center protein L	-5.972	0.00031
Alpha-(1->3)-arabinofuranosyltransferase	-5.963	0.00031
Serine/threonine-protein kinase F	-5.943	0.00031

E

**Gene**	**FDR-Adj. P**	**NES**
Thymidylate synthase 1	-5.522	0.00055
DNA-binding protein Bv3F	-5.446	0.00055
mupirocin-resistant isoleucine–tRNA ligase MupA	-5.294	0.00055
Cobalt-precorrin-7 C(5)-methyltransferase	-5.114	0.00055
Cobalt-zinc-cadmium resistance protein CzcI	-5.103	0.00055
IS200/IS605 family transposase ISCth10	-4.897	0.00055
Na(+)-translocating ferredoxin:NAD(+) oxidoreductase complex subunit C	-4.843	0.00055
Anaerobic sulfite reductase subunit C	-4.814	0.00055
(R)-2-hydroxyglutaryl-CoA dehydratase activating ATPase	-4.812	0.00055
Salicylate 5-hydroxylase, large oxygenase component	-4.778	0.00055
Tyrosine aminotransferase	-4.765	0.00055
putative protein YgcP	-4.703	0.00055
Alkaline phosphatase PhoK	-4.685	0.00055
Salicylate 5-hydroxylase, small oxygenase component	-4.684	0.00055
Propanediol utilization protein PduU	-4.64	0.00055
Putative superoxide reductase	-4.596	0.00055

Sortase B	-4.552	0.00055
Propanediol utilization protein PduV	-4.424	0.00055
Elongation factor G, mitochondrial	-4.343	0.00055
Germination protease	-4.315	0.00055
Stage IV sporulation protein A	-4.315	0.00055
Stage V sporulation protein AD	-4.315	0.00055
putative N-glycosylase/DNA lyase	-4.309	0.00055
(R)-phenyllactyl-CoA dehydratase alpha subunit	-4.297	0.00055
Nickel-cobalt-cadmium resistance protein NccX	-4.295	0.00055
(R)-2-hydroxyglutaryl-CoA dehydratase, subunit beta	-4.293	0.00055
Putative transport protein YbjL	-4.292	0.00055
RNA polymerase sigma-G factor	-4.286	0.00055
Translocation-enhancing protein TepA	-4.271	0.00055
Stage V sporulation protein T	-4.261	0.00055
Light-activated DNA-binding protein EL222	-4.254	0.00055
RNA polymerase sigma-28 factor	-4.245	0.00055
putative anti-sigma-F factor NrsF	-4.242	0.00055
Oxalate-binding protein	-4.225	0.00055
IS256 family transposase ISCth4	-4.222	0.00055
Propanediol utilization protein PduB	-4.202	0.00055
Stage III sporulation protein D	-4.195	0.00055
Spore protein YabP	-4.194	0.00055
3,4-dehydroadipyl-CoA semialdehyde dehydrogenase	-4.18	0.00055
Nickel and cobalt resistance protein CnrR	-4.118	0.00055
Neopullulanase 1	-4.117	0.00055
Small, acid-soluble spore protein C2	-4.111	0.00055
Mini-ribonuclease 3-like protein	-4.103	0.00055
L-threonine kinase	-4.09	0.00055
IS3 family transposase ISStma17	-4.039	0.00055
Outer membrane protein 40	-4.031	0.00055
Methionine-rich peptide X	-3.993	0.00055
IS5 family transposase ISBmu20	-3.97	0.00055
Reverse rubrerythrin-1	-3.967	0.00055
Histidine racemase	-3.951	0.00055
Spore germination protein B1	-3.947	0.00055
2-pyrone-4,6-dicarboxylate hydrolase	-3.936	0.00055
Glycine/sarcosine/betaine reductase complex component C subunit alpha	-3.931	0.00055
Tryptophanase 1	-3.919	0.00055
Nickel and cobalt resistance protein CnrC	-3.918	0.00055
putative sporulation protein YlmC	-3.91	0.00055
mupirocin-resistant isoleucine–tRNA ligase MupB	-3.907	0.00055
putative deoxyuridine 5’-triphosphate nucleotidohydrolase YncF	-3.89	0.00055
IS110 family transposase ISCaa14	-3.886	0.00055

SpoIVB peptidase	-3.885	0.00055
Glycine reductase complex component B subunit gamma	-3.874	0.00055
Propionate catabolism operon regulatory protein	-3.87	0.00055
RNA polymerase sigma-35 factor	-3.865	0.00055
Propanediol dehydratase medium subunit	-3.852	0.00055
Chloroacetanilide N-alkylformylase, ferredoxin reductase component	-3.835	0.00055
Ribulose bisphosphate carboxylase large chain, chromosomal	-3.835	0.00055
IS66 family transposase ISBcen14	-3.824	0.00055
putative tryptophan transport protein	-3.82	0.00055
IS66 family transposase ISBcen19	-3.809	0.00055
Peptidoglycan-N-acetylmuramic acid deacetylase PdaA	-3.808	0.00055
Light-harvesting protein B-870 beta chain	-3.798	0.00055
PEP-dependent dihydroxyacetone kinase 2, phosphoryl donor subunit DhaM	-3.796	0.00055
Glycine reductase complex component B subunits alpha and beta	-3.793	0.00055
IS1182 family transposase ISCpe5	-3.79	0.00055
Accessory gene regulator protein B	-3.788	0.00055
CRISPR-associated endoribonuclease Cas6	-3.787	0.00055
Phosphoglycolate phosphatase, plasmid	-3.786	0.00055
Glycerol dehydratase large subunit	-3.781	0.00055
Glycine/sarcosine/betaine reductase complex component C subunit beta	-3.773	0.00055
Diol dehydratase-reactivating factor alpha subunit	-3.759	0.00055
IS3 family transposase ISElsp1	-3.754	0.00055
Stage II sporulation protein E	-3.754	0.00055
IS110 family transposase ISCaa7	-3.754	0.00055
Propanediol dehydratase small subunit	-3.748	0.00055
Reverse rubrerythrin-2	-3.747	0.00055
Cytochrome c-type protein SHP	-3.744	0.00055
Antigen TpF1	-3.731	0.00055
Serine/threonine-protein kinase CtkA	-3.731	0.00055
Diadenosine hexaphosphate hydrolase	-3.73	0.00055
Glycine/sarcosine/betaine reductase complex component A	-3.724	0.00055
Outer membrane protein 41	-3.721	0.00055
Stage III sporulation protein AE	-3.703	0.00055
N-acetylmuramoyl-L-alanine amidase	-3.7	0.00055
Catechol 1,2-dioxygenase 2	-3.696	0.00055
Glycine/sarcosine/betaine reductase complex component A1	-3.678	0.00055
D-proline reductase proprotein PrdA	-3.654	0.00055
Catechol 1,2-dioxygenase 1	-3.633	0.00055
Phthalate 4,5-dioxygenase oxygenase reductase subunit	-3.63	0.00055
Iron hydrogenase 1	-3.623	0.00055
Metal-staphylopine import system ATP-binding protein CntD	-3.618	0.00055

F

Gene	FDR-Adj. P	NES
Bifunctional protein MdtA	-5.039	0.00064
Flagellar assembly protein FliX	-4.91	0.00064
Presqualene diphosphate synthase	-4.54	0.00064
mupirocin-resistant isoleucine–tRNA ligase MupA	-4.537	0.00064
Formyltransferase/hydrolase complex subunit D	-4.476	0.00064
Formyltransferase/hydrolase complex Fhc subunit A	-4.455	0.00064
Methenyltetrahydromethanopterin cyclohydrolase	-4.406	0.00064
3',5'-cyclic-nucleotide phosphodiesterase	-4.329	0.00064
Formyltransferase/hydrolase complex Fhc subunit C	-4.301	0.00064
Methylmalonyl-CoA mutase small subunit	-4.277	0.00064
Bifunctional dihydropteroate synthase/dihydropteroate reductase	-4.27	0.00064
Plasminogen-binding protein PgbB	-4.237	0.00064
Oxygen-independent coproporphyrinogen-III oxidase-like protein HemZ	-4.205	0.00064
GTP cyclohydrolase 1 type 2	-4.195	0.00064
5,6,7,8-tetrahydromethanopterin hydro-lyase	-4.19	0.00064
Methanol dehydrogenase [cytochrome c] subunit 2	-4.181	0.00064
Sensor protein DivL	-4.173	0.00064
Hydroxycarboxylate dehydrogenase B	-4.148	0.00064
Sortase B	-4.142	0.00064
2-amino-5-chloromuconate deaminase	-4.127	0.00064
L-hydantoinase	-4.024	0.00064
Cytochrome c-L	-4.022	0.00064
Flagellar FliL protein	-4.012	0.00064
Putative ATP-dependent DNA helicase YjcD	-3.986	0.00064
Beta-methylmalyl-CoA dehydratase	-3.968	0.00064
Cytochrome c-553	-3.959	0.00064
(2R)-sulfolactate sulfo-lyase subunit alpha	-3.955	0.00064
Malyl-CoA/beta-methylmalyl-CoA/citramalyl-CoA lyase	-3.953	0.00064
Oxalate:formate antiporter	-3.934	0.00064
Inducible ornithine decarboxylase	-3.921	0.00064
Dihydromethanopterin reductase	-3.891	0.00064
10 kDa chaperonin 2	-3.884	0.00064
Na(+)-translocating ferredoxin:NAD(+) oxidoreductase complex subunit G	-3.882	0.00064
Lipoprotein NlpI	-3.873	0.00064
Bifunctional DNA-directed RNA polymerase subunit beta-beta'	-3.83	0.00064
Inner membrane protein YabI	-3.823	0.00064
Cbb3-type cytochrome c oxidase subunit FixP	-3.797	0.00064
Bifunctional coenzyme PQQ synthesis protein C/D	-3.774	0.00064
Opacity-associated protein OapA	-3.769	0.00064
Surface-adhesin protein E	-3.769	0.00064
Accessory gene regulator protein B	-3.752	0.00064
Blue-light absorbing proteorhodopsin	-3.709	0.00064

Beta-(1–>2)glucan export ATP-binding/permease protein NdvA	-3.708	0.00064
Chaperone protein YcdY	-3.702	0.00064
Ribulose bisphosphate carboxylase large chain 2	-3.684	0.00064
Outer membrane protein P5	-3.68	0.00064
Chloramphenicol resistance protein CraA	-3.654	0.00064
Na(+)-translocating ferredoxin:NAD(+) oxidoreductase complex subunit C	-3.644	0.00064
Neopullulanase 1	-3.64	0.00064
DNA transformation protein TfoX	-3.632	0.00064
Beta-carotene 15,15’-dioxygenase	-3.631	0.00064
Redox-sensing transcriptional repressor Rex 1	-3.631	0.00064
Metallopeptidase AprA	-3.629	0.00064
Rubrerythrin-1	-3.628	0.00064
Hydrogenase/urease maturation factor HypB	-3.615	0.00064
Small, acid-soluble spore protein C2	-3.598	0.00064
Ribulose bisphosphate carboxylase small chain 2	-3.595	0.00064
Translocation-enhancing protein TepA	-3.591	0.00064
Gamma-glutamyl-L-1-hydroxyisopropylamide hydrolase	-3.587	0.00064
putative protein YgcP	-3.566	0.00064
RNA polymerase sigma-28 factor	-3.526	0.00064
PTS system N-acetylglucosamine-specific EIIB component	-3.52	0.00064
Na(+)-translocating ferredoxin:NAD(+) oxidoreductase complex subunit D	-3.513	0.00064
Germination protease	-3.511	0.00064
Stage IV sporulation protein A	-3.511	0.00064
Squalene–hopene cyclase	-3.508	0.00064
putative cobalt-factor III C(17)-methyltransferase	-3.491	0.00064
USG-1 protein	-3.485	0.00064
Valine dehydrogenase	-3.482	0.00064
Molybdenum storage protein subunit alpha	-3.479	0.00064
Stage V sporulation protein AD	-3.476	0.00064
RNA polymerase sigma-G factor	-3.474	0.00064
5-(methylthio)ribulose-1-phosphate aldolase	-3.473	0.00064
Translational regulator CsrA2	-3.469	0.00064
Spore protein YabP	-3.459	0.00064
Stage V sporulation protein T	-3.446	0.00064
Oxalyl-CoA decarboxylase	-3.444	0.00064
Quinone-reactive Ni/Fe-hydrogenase large chain	-3.442	0.00064
Translational regulator CsrA1	-3.44	0.00064
L-proline trans-4-hydroxylase	-3.437	0.00064
Undecaprenyl-diphosphooligosaccharide–protein glycotransferase	-3.428	0.00064
D(-)-tartrate dehydratase	-3.423	0.00064
Resuscitation-promoting factor Rpf	-3.42	0.00064
Nucleoid-associated protein Lsr2	-3.405	0.00064
Elongation factor G, mitochondrial	-3.399	0.00064

Potassium/sodium uptake protein NtpJ	-3.398	0.00064
Malate synthase	-3.396	0.00064
Formyltransferase/hydrolase complex Fhc subunit B	-3.394	0.00064
Stage III sporulation protein D	-3.381	0.00064
Putative septation protein SpoVG	-3.369	0.00064
Hemolysin C	-3.365	0.00064
Tyrosine recombinase XerH	-3.355	0.00064
NAD(P)-dependent methylenetetrahydromethanopterin dehydrogenase	-3.355	0.00064
Oxalate decarboxylase OxdD	-3.353	0.00064
Cobalt-dependent inorganic pyrophosphatase	-3.352	0.00064
60 kDa chaperonin 3	-3.351	0.00064
Protein PhoH	-3.35	0.00064
Glutathione amide-dependent peroxidase	-3.342	0.00064
putative quinol monooxygenase YgiN	-3.326	0.00064
DNA-binding protein HB1	-3.326	0.00064

G

**Gene**	**FDR-Adj. P**	**NES**
Sirohydrochlorin cobaltochelatase CbiKP	5.05	0.00064
Bifunctional protein MdtA	4.982	0.00064
Cytochrome c-L	4.843	0.00064
Methanol dehydrogenase [cytochrome c] subunit 2	4.798	0.00064
Carbon monoxide dehydrogenase 1	4.71	0.00064
NAD(+)–dinitrogen-reductase ADP-D-ribosyltransferase	4.631	0.00064
Carbon monoxide dehydrogenase/acetyl-CoA synthase subunit alpha	4.487	0.00064
Corrinoid/iron-sulfur protein large subunit	4.47	0.00064
Hydrogenase-2 large chain	4.455	0.00064
Molybdenum storage protein subunit beta	4.406	0.00064
Sulfite reductase, dissimilatory-type subunit gamma	4.308	0.00064
Acetolactate synthase isozyme 1 small subunit	4.265	0.00064
Protein DsvD	4.254	0.00064
mupirocin-resistant isoleucine–tRNA ligase MupA	4.214	0.00064
Hopanoid C-3 methylase	4.162	0.00064
Menaquinone reductase, iron-sulfur cluster-binding subunit	4.161	0.00064
Metal-binding protein SmbP	4.145	0.00064
Menaquinone reductase, molybdopterin-binding-like subunit	4.065	0.00064
Reverse rubrerythrin-1	4.054	0.00064
Rubredoxin 3	4.026	0.00064
Hydrogenase-2 small chain	3.991	0.00064
Ribulose bisphosphate carboxylase small chain 2	3.969	0.00064
Periplasmic [NiFe] hydrogenase large subunit	3.965	0.00064
Ribulose bisphosphate carboxylase large chain 2	3.918	0.00064
Menaquinone reductase, multiheme cytochrome c subunit	3.913	0.00064

(R)-2-hydroxyisocaproyl-CoA dehydratase beta subunit	3.895	0.00064
Formyltransferase/hydrolase complex Fhc subunit B	3.822	0.00064
Toluene-4-monooxygenase system, ferredoxin component	3.812	0.00064
Hydroxylamine oxidoreductase	3.79	0.00064
Menaquinone reductase, integral membrane subunit	3.777	0.00064
Accessory gene regulator protein B	3.775	0.00064
Resuscitation-promoting factor Rpf	3.76	0.00064
Split-Soret cytochrome c	3.752	0.00064
Carbon monoxide dehydrogenase 2	3.747	0.00064
Dihydromethanopterin reductase	3.726	0.00064
Protein FeSII	3.712	0.00064
PTS system N-acetylglucosamine-specific EIIB component	3.705	0.00064
Nitrogen fixation regulatory protein	3.626	0.00064
Na(+)-translocating ferredoxin:NAD(+) oxidoreductase complex subunit C	3.608	0.00064
Alpha-amylase 1	3.584	0.00064
Elongation factor G, mitochondrial	3.576	0.00064
Neopullulanase 1	3.574	0.00064
Hydrogenase-4 component G	3.525	0.00064
Ribulose bisphosphate carboxylase	3.525	0.00064
D-xylonate dehydratase YagF	3.51	0.00064
Sporulation-specific cell division protein SsgB	3.506	0.00064
EtfAB:quinone oxidoreductase	3.505	0.00064
Lipoprotein NlpI	3.494	0.00064
CRISPR-associated endonuclease Cas6	3.461	0.00064
Cytochrome c-type protein ImcH	3.421	0.00064
Opacity-associated protein OapA	3.417	0.00064
Surface-adhesin protein E	3.417	0.00064
Fused nickel transport protein NikMN	3.416	0.00064
Molybdenum storage protein subunit alpha	3.414	0.00064
Corrinoid/iron-sulfur protein small subunit	3.399	0.00064
Small, acid-soluble spore protein C2	3.399	0.00064
Cytochrome c”	3.384	0.00064
IS1182 family transposase ISRssp12	3.375	0.00064
Mannosylglucosyl-3-phosphoglycerate synthase	3.347	0.00064
Flagellar FliL protein	3.344	0.00064
Valine dehydrogenase	3.333	0.00064
Sensor protein CseC	3.319	0.00064
Tyrosine-protein kinase CpsD	3.312	0.00064
Rubredoxin-oxygen oxidoreductase	3.306	0.00064
putative nitrate/nitrite transporter NarK2	3.298	0.00064
5-hydroxybenzimidazole synthase BzaA	3.297	0.00064
Enoyl-[acyl-carrier-protein] reductase [NADPH] FabI	3.295	0.00064
Putative sulfur carrier protein YeeD	3.292	0.00064

Translocation-enhancing protein TepA	3.28	0.00064
IS66 family transposase ISSwo2	3.258	0.00064
Citrate (Re)-synthase	3.257	0.00064
putative protein YgcP	3.257	0.00064
PTS system N-acetylglucosamine-specific EIIC component	3.256	0.00064
Putative superoxide reductase	3.256	0.00064
Cyanuric acid amidohydrolase	3.247	0.00064
IS91 family transposase ISCARN110	3.24	0.00064
IS5 family transposase ISPso2	3.234	0.00064
IS1595 family transposase ISMpo2	3.232	0.00064
60 kDa chaperonin 3	3.229	0.00064
Particulate methane monooxygenase beta subunit	3.206	0.00064
Oxalate oxidoreductase subunit beta	3.205	0.00064
10 kDa chaperonin 2	3.2	0.00064
Sucrose synthase	3.199	0.00064
putative sporulation protein YlmC	3.187	0.00064
IS66 family transposase ISDpr4	3.186	0.00064
DNA transformation protein TfoX	3.185	0.00064
(R)-2-hydroxyisocaproyl-CoA dehydratase alpha subunit	3.17	0.00064
Outer membrane protein P5	3.159	0.00064
putative secretion system apparatus ATP synthase SsaN	3.15	0.00064
IS1182 family transposase ISClbu1	3.15	0.00064
2-amino-5-chloromuconate deaminase	3.147	0.00064
Type A flavoprotein fprA	3.145	0.00064
Benzylsuccinate synthase activating enzyme	3.144	0.00064
(R)-phenyllactate dehydratase activator	3.142	0.00064
Particulate methane monooxygenase alpha subunit	3.133	0.00064
(R)-phenyllactyl-CoA dehydratase alpha subunit	3.126	0.00064
Barbiturase 1	3.122	0.00064
CRISPR system Cascade subunit CasE	3.112	0.00064
NADH-dependent phenylglyoxylate dehydrogenase subunit gamma	3.106	0.00064
ECF RNA polymerase sigma factor ShbA	3.103	0.00064
